# Bacterial and fungal characterization of pancreatic adenocarcinoma from Endoscopic Ultrasound-guided biopsies

**DOI:** 10.3389/fimmu.2023.1268376

**Published:** 2023-10-13

**Authors:** Robin D. Wright, Thais F. Bartelli, Seyda Baydogan, James Robert White, Michael P. Kim, Manoop S. Bhutani, Florencia McAllister

**Affiliations:** ^1^ Department of Clinical Cancer Prevention, The University of Texas MD Anderson Cancer Center, Houston, TX, United States; ^2^ Department of Surgical Oncology, The University of Texas MD Anderson Cancer Center, Houston, TX, United States; ^3^ Department of Clinical Cancer Genetics, The University of Texas MD Anderson Cancer Center, Houston, TX, United States; ^4^ Department of Gastroenterology, Hepatology and Nutrition, The University of Texas MD Anderson Cancer Center, Houston, TX, United States; ^5^ Department of Gastrointestinal Medical Oncology, The University of Texas MD Anderson Cancer Center, Houston, TX, United States; ^6^ Department of Immunology, The University of Texas MD Anderson Cancer Center, Houston, TX, United States

**Keywords:** tumor microbes, pancreatic cancer, endoscopic ultrasound, fine needle aspiration, microbiome, pancreatic adenocarcinoma

## Abstract

**Introduction:**

The tumor microbiome (TM) has been linked to pancreatic cancer prognosis. Specific microbes can confer tumor resistance to therapies. Early knowledge of the TM at time of diagnosis would be clinically relevant for precision therapy based on microbial composition. However, it is difficult to define the TM prior to surgical resection.

**Methods:**

In this pilot feasibility study, patients underwent Endoscopic Ultrasound-Fine Needle Aspiration (EUS-FNA) biopsy of pancreatic adenocarcinoma. These samples were analyzed using 16S rRNA and internal transcribed spacer (ITS) sequencing for characterization of the tumor bacteria and fungi.

**Result:**

After in silico decontamination and comparison to non-matched tumor, we were able to characterize the TM in biopsies, which was comparable to the TM from surgical specimens.

**Discussion:**

EUS-FNA biopsy may represent a feasible modality to characterize the pancreatic TM prior to surgical resection with proper decontamination strategies and improvements in matched controls.

## Introduction

There were an estimated 60,000 new cases and approximately 50,000 deaths due to pancreatic cancer (PC) in 2022 (1). Pancreatic ductal adenocarcinoma (PDAC) remains one of the most lethal of all cancers and is estimated to be the second highest cause of cancer-related mortality by 2030 (2). Endoscopic ultrasound (EUS) is used to evaluate the pancreas via transgastric and transduodenal imaging in high-risk individuals undergoing regular screening or in patients with a mass detected by other methods to confirm diagnosis (3). Samples are frequently taken for cytological analysis via fine needle aspiration (FNA) or histological assessment with core needle biopsy.

There has been an increase in the understanding of the gut and tumor microbiomes (TM) in cancer and their significant role in disease progression and responses to therapy (4–6). As a potential diagnostic tool, the gut microbiome was shown to be capable of identifying cases of PC amongst healthy controls and other benign diseases of the pancreas (7, 8). It was also determined that distinct TM signatures could be found between short- and long-term survivors of PC (4). In addition to the potential utility for diagnosis and determining prognostic factors, modulation of the gut and TM for greater immune activation and leverage against cancer has been postulated (9). PC is a heterogeneous disease process and precision oncology approaches will increasingly be required in the treatment of PC (10). In addition, manipulation of the TM by fecal microbial transplant is currently being investigated as a treatment modality against PC (NCT04975217). While the gut microbiome can be easily characterized, there is a deficiency of methods to detect the pancreatic TM prior to a surgical resection. Here we present the results of a pilot study to test feasibility of assessing bacteria and fungal populations in pancreatic tumors via EUS-FNA biopsy.

## Methods

### Specimen retrieval

Both 16s and ITS sequencing were performed on samples (*n*=5) obtained by use of Endoscopic Ultrasound and Fine Needle Aspiration biopsy (EUS-FNA biopsy) in individuals that underwent examination at M. D. Anderson Cancer Center (USA). Specimens were obtained under the informed consent of the MD Anderson Cancer Center (MDACC) IRB-approved protocol PA16-0911v07 (Analysis of the Microbiome in Patients with GI Cancer, at High Risk for GI Cancer, and Controls). Patients with known or suspected PDAC who were undergoing a clinically indicated EUS for fine needle aspiration cytology/biopsy and/or fiducials underwent two core biopsies of the pancreatic tumor for microbiome sequencing using Procore (Cook) 25G (n=4) and 22G (n=1) core biopsy needles using a transgastric (n=4) or transduodenal (n=1) approach. One of the five patients had received neoadjuvant chemotherapy (Gemcitabine/Abraxane) while the other four were treatment-naïve. Core biopsies were flash frozen, transferred in liquid nitrogen, and stored at -80 degrees Celsius until the time of DNA extraction. Patient surgical tumor samples were collected only after planned surgical resection and pathologic examination. Samples were similarly flash frozen, transferred in liquid nitrogen and stored at -80 degrees Celsius until DNA extraction. A total of 10 patient samples were obtained from pancreatectomies performed for PC from 2009 to 2019. The surgical tissue samples were collected under the Pancreas Tissue Bank, LAB00-396, and subsequently analyzed under a research-use protocol (PA16-0911).

### Microbiome characterization

Sample DNAs were extracted with RNeasy Powerlyzer Tissue and Cell Kit (Qiagen, Cat. Number 69516) automated on the QIAcube Connect instruments. 16S rRNA and ITS2 gene sequencing methods were adapted from those developed for the NIH-Human Microbiome Project and the Earth Microbiome Project (11, 12). The 16S rDNA region v4 was amplified by polymerase chain reaction (PCR) and sequenced on the Illumina MiSeq platform in a 2x250 base pair paired-end protocol. The primers used for amplification contain adapters for MiSeq sequencing and single-index barcodes so that the PCR products may be pooled and sequenced directly, targeting at least 10,000 reads per sample. Raw paired-end 16S rRNA and ITS reads were merged into consensus fragments by FLASH (13) and subsequently filtered for quality (targeted error rate < 0.5%) and length (minimum 200bp) using Trimmomatic (14) and QIIME (15, 16). Spurious hits to the PhiX control genome were identified using BLASTN and removed. Passing sequences were trimmed of primers, evaluated for chimeras with UCLUST (17), and screened for human-associated contaminant using Bowtie2 (18). Chloroplast and mitochondrial contaminants were detected and filtered using the RDP classifier with a confidence threshold of 50% (19). High-quality passing 16S rRNA sequences were assigned to a high-resolution taxonomic lineage using Resphera Insight (20, 21). High-quality passing ITS sequences were clustered into OTUs by UCLUST (*de novo* mode) and assigned a taxonomic lineage using the RDP classifier with the UNITE database. Data analysis was carried out in R studio 2023.03.0, with the appropriate packages (plyr, ggplot). Venn diagram was performed with InteractiVenn (22).

**Figure 1:**
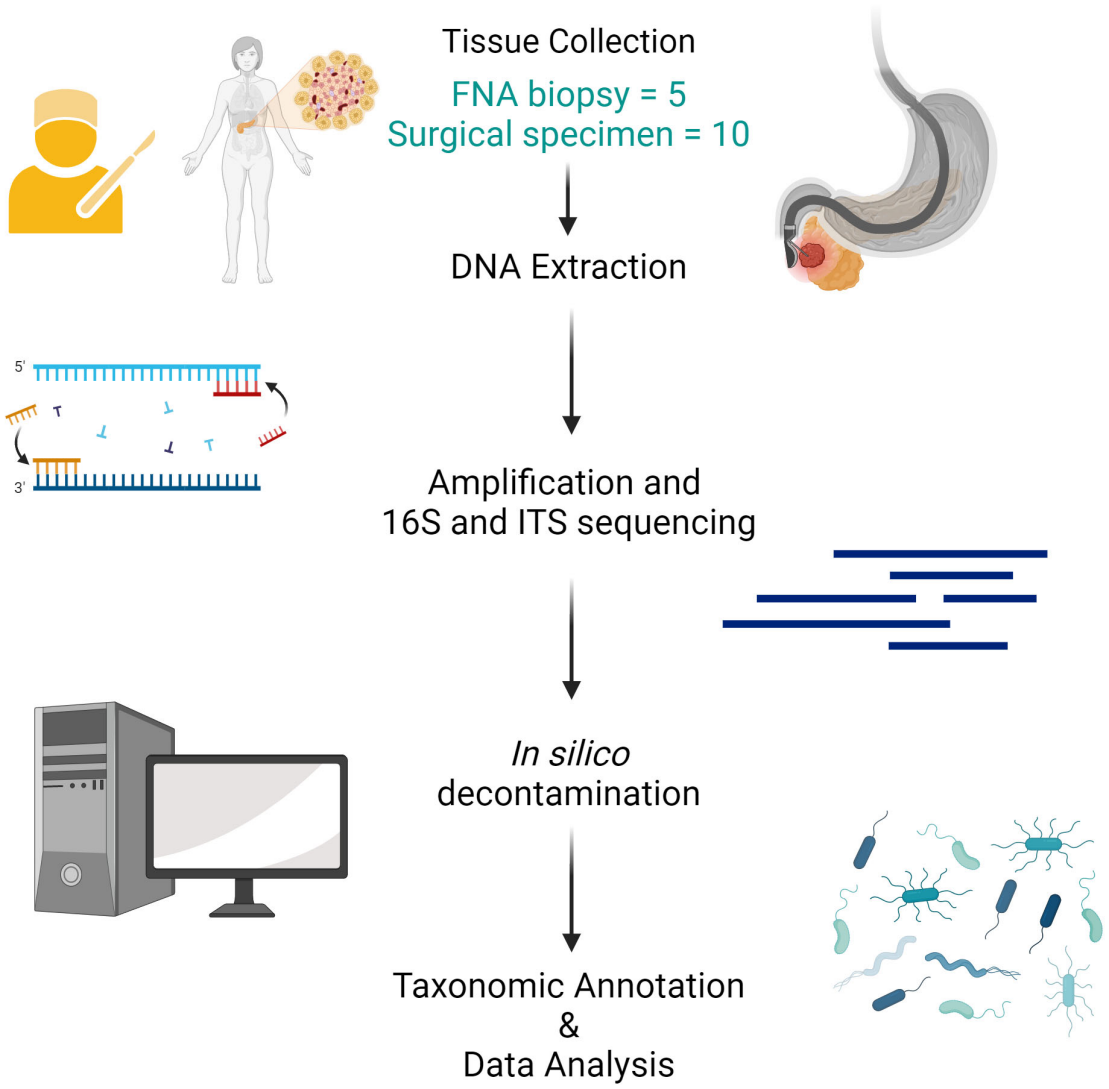
Workflow schema.

Due to the passage of the endoscope per-orally through the esophagus and stomach and the nature of EUS-FNA biopsy (transgastric or transduodenal), there is a high propensity to be biased by microbial contaminants as well as intrinsic biases associated with 16S and ITS sequencing methods. In order to remove spurious genera commonly found as contaminants in 16S rRNA sequencing due to manipulation of samples and/or those commonly found as reagents and laboratory contaminants, we relied on curated exclusion lists previously published (4, 23) with supplementary correlation analysis against contaminant taxa to identify bacteria or fungi for exclusion. For fungi, contaminant removal was based on the identification of *de novo* OTUs rather than an exclusion list, and those with Spearman correlations exceeding 0.3 against any indicator contaminant OTU were also removed. Additional analysis also identified fungal genera such as *Malassezia, Cystidiodontia* and, *Cladosporum* as possible contaminants and they were later excluded (**Supplementary Table S1**). After contaminant filtering, 65 and 19 bacterial and fungal genera, respectively, remained in the analysis (**Figure 1**).

## Results

**Figure 2:**
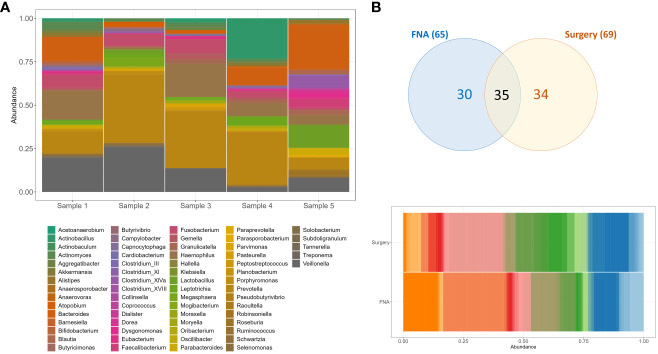
Bacteria genera composition of unmatched pancreatic FNA and surgical tissue samples obtained from individuals with PC. **(A)** Genera composition of bacteria per FNA sample. **(B)** Venn diagram and barplot of genera composition of surgical specimens (*n*=10) and FNA samples (*n*=5). Sample DNAs were extracted with RNeasy Powerlyzer Tissue and Cell Kit and region V4 (16s rRNA) sequenced by Illumina MiSeq v2 2x250 v1.8.

At least seven bacterial genera (11%) were identified in all five samples sequenced (*Actinomyces, Campylobacter, Fusobacterium, Granulicatella, Haemophilus, Prevotella*, and *Veilonella*), with *Prevotella* as the most abundant (**Figure 2A**). When compared to fresh frozen surgical pancreatic tissue from unmatched individuals with PC (*n=* 10), we observed at least 35 (54%) genera in common between these two sample types (**Figure 2B**). This similarity is noteworthy considering that the surgical specimens were obtained from different individuals and matched irrespective of the anatomical portion of the pancreas or the collection moment (pre or post-chemotherapy treatment).

Among the unique genera (*n*=30) identified in the FNA biopsy samples we cannot disregard that some or most of them might represent bacteria from the oropharynx, esophagus, stomach or duodenum that could contaminate the endoscope and the pancreatic biopsy tissue during the procedure and the route used. Previous studies have identified *Oribacterium, Atopobium, Klebsiella, Butyvibrio, Pasteurella, Tanerella*, and *Coprococcus* in the mucosa derived from either duodenum or stomach samples (24–27). Other genera such as *Fusobacterium, Haemophilus, Actinobacillus, Eubacterium, Actinobaculum, Bifidobacterium, Blautia, Veilonella, Prevotella* and *Lactobacillus* were also identified in duodenum and stomach samples but were found in the FNA and the pancreatic surgical specimens as well (**Supplementary Table S2**) (24–27). These results are consistent with the notion that the human pancreas can share microbiota composition with gastrointestinal sites and ultimately the differential abundance of certain bacteria may be as relevant to consider as solely their presence or absence. The use of proper negative controls, amplification negative controls, and matched duodenum or stomach mucosa microbiome examination will help future decontamination strategies.

Recently, Nakano et al. (28) described the predominance of *Acinetobacter* and *Pseudomonas* in the pancreatic FNA samples in comparison to the duodenum and/or stomach mucosa from matched individuals, while in our study and many others (14, 29–31), these two genera were included on the putative contaminants list and were therefore excluded from the analysis. Additionally, these FNA samples were not compared to a matched resected tumor and it was not described whether the genera identified were solely in each sample type or shared among them (pancreatic FNA, mucosal duodenum, or stomach) (28). Similarly, Masi et al. (32) previously investigated the feasibility of using pancreatic formalin-fixed paraffin-embedded (FFPE) samples from a small number of EUS procedures to study the pancreatic microbiome and identified the existence of significant differences in the overall bacterial composition of the samples according to the route used for examination (duodenum or stomach), with *Fusobacterium* significantly enriched when the stomach route was used. In this sense, there is still a need to better comprehend the potential of the FNA pancreatic microbiome as a diagnostic tool for PC, including the bacterial composition of surrounding tissues and putative contaminants.

**Figure 3:**
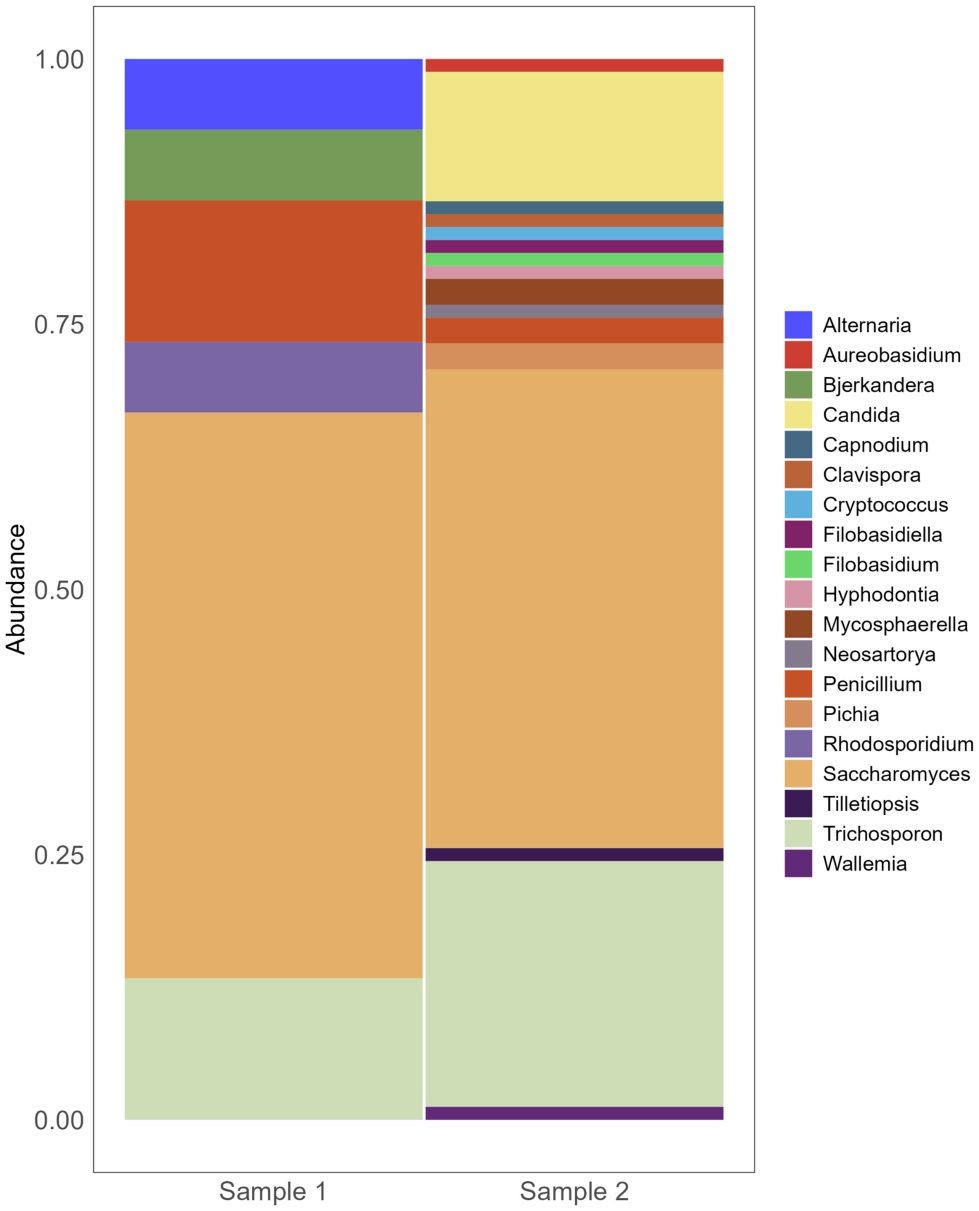
Fungi composition of pancreatic FNA obtained from individuals with PC. Barplot of genera composition per FNA sample individually (*n*=2). After DNAs extraction, ITS2 got sequenced by Illumina MiSeq v2 2x250 v1.8.

Additionally, in our cohort, two out of the five patients that underwent 16S sequencing successfully had their FNA fungal composition determined by ITS, supporting the efficacy of these minimally invasive endoscopically collected samples for complementary fungi-bacteria signature assessment. Despite the reduced number of FNAs tested, we observed relevant similarities in their fungal population even after decontamination, with high prevalence of *Saccharomyces* and *Trichosporon* (**Figure 3**). As most genera remain unknown for PC, there is a need to overcome experimental biases *in vivo* and *silico* associated with fungal identification by ITS sequencing in tumors.

## Discussion

We report the successful characterization of the pancreatic microbiome using EUS-FNA biopsy, including the first report of ITS sequencing from EUS-FNA biopsy samples. This method of microbial biopsy could be explored for use in diagnosis and prognosis. It has become the standard of practice to perform next generation sequencing (NGS) in all patients diagnosed with PC for precision oncology approaches (33). In the future, monitoring and manipulating the TM could expand individualized treatments with the understanding that certain bacterial signatures are associated with a favorable prognosis (4).

Alam et al. found that in mice, the intratumoral mycobiome of pancreatic lesions affects secretion of IL-33 which in turn induces type 2 immunity in pancreatic cancer. Both anti-fungal treatment or IL-33 suppression decreased T_H_2 infiltration and caused tumor regression (34, 35). These findings suggest alterations to the fungi, or mycobiome, in addition to intratumoral bacteria, may be another avenue of precision therapy.

Previously, duodenal fluid has been used as a surrogate marker for the pancreatic microbiome and it was found that patients with PDAC had lower microbial diversity in their duodenal fluid than controls with a normal pancreas (36). Techniques to accurately sample the microbiome of pancreatic lesions are urgently needed. Devices are being developed in order to meet this need (37), but conventional EUS-FNA biopsy may be a sufficient tool. It is also theoretically possible to perform immunoprofiling on the same EUS-FNA biopsy samples that are used to characterize the TM. Analysis of the microbiome and immune profile, in addition to the currently recommended NGS, could further inform directed therapies through multi-omic strategies.

We describe a characterization of the microbiome of pancreatic lesions using EUS-FNA biopsy combined with *in silico* decontamination. Our bacterial signature was conserved when compared to non-matched pancreatic tissue. We also used ITS sequencing to identify fungal species. Within a host-tissue background, direct sequencing of 16S rRNA and ITS amplicons offers a more direct and sensitive approach to assess microbial composition than whole-genome sequencing (WGS) or RNA-seq datasets, which are often dominated by host genomic DNA. Though samples are more routinely being taken for NGS in EUS-FNA for pancreatic lesions, the authors do not suggest extrapolating microbiome characterization from WGS data for the aforementioned reasons.

This proof of concept method could be utilized in the identification of the microbiome and mycobiome of pancreatic lesions prior to resection. Further investigation is needed with greater samples sizes, using mucosal tissue for normalization, and matched tissues for validation.

## Data availability statement

The data presented in the study are deposited in the SRA under accession PRJNA1008674. The data can be found here https://www.ncbi.nlm.nih.gov/sra/?term=PRJNA1008674.

## Ethics statement

The studies involving humans were approved by University of Texas MD Anderson Cancer Center- Institutional Review Board. The studies were conducted in accordance with the local legislation and institutional requirements. The participants provided their written informed consent to participate in this study.

## Author contributions

RW: Conceptualization, Writing – original draft, Writing – review & editing. TB: Investigation, Methodology, Visualization, Conceptualization, Writing – original draft, Data curation, Formal Analysis. SB: Validation, Conceptualization, Formal Analysis, Investigation, Methodology, Visualization, Writing – original draft. JW: Data curation, Software, Writing – review & editing, Methodology, Validation, Visualization. MK: Investigation, Resources, Supervision, Writing – review & editing. MB: Conceptualization, Funding acquisition, Visualization, Writing – original draft, Investigation, Resources, Supervision, Writing – review & editing. FM: Methodology, Project administration, Validation, Conceptualization, Funding acquisition, Investigation, Supervision, Visualization, Writing – original draft, Writing – review & editing.
