# Supplementary material for: Bacterial and fungal characterization of pancreatic adenocarcinoma from Endoscopic Ultrasound-guided biopsies
Source: Front Immunol. 2023 Oct 13;14:1268376. doi: 10.3389/fimmu.2023.1268376 (PMC10611524; doi:10.3389/fimmu.2023.1268376)
Supplement: Supplementary file 1 [file Table_1.docx]

**Table S1.** List of identified bacterial contaminants.

| Bacteria | | | |
| --- | --- | --- | --- |
| *Achromobacter* | *Delftia* | *Micrococcus* | *Roseomonas* |
| *Aciditerrimonas* | *Dietzia* | *Mitsuaria* | *Rothia* |
| *Acidovorax* | *Enhydrobacter* | *Mycobacterium* | *Rugamonas* |
| *Acinetobacter* | *Ensifer* | *Neisseria* | *Schlegelella* |
| *Afipia* | *Enterobacter* | *Novoherbaspirillum* | *Shewanella* |
| *Agrobacterium* | *Escherichia* | *Oceanospirillales* | *Shigella* |
| *Aquabacterium* | *Francisella* | *Oxalicibacterium* | *Sphingobium* |
| *Azoarcus* | *Halobacteriaceae* | *Oxalobacteraceae* | *Sphingomonadaceae* |
| *Bacillus* | *Halomonadaceae* | *Paenibacillus* | *Sphingomonas* |
| *Bradyrhizobium* | *Herbaspirillum* | *Pedobacter* | *Staphylococcus* |
| *Burkholderia* | *Holophagaceae* | *Pelomonas* | *Stenotrophomonas* |
| *Burkholderiaceae* | *Kiloniella* | *Phyllobacterium* | *Streptococcus* |
| *Caldimonas* | *Kocuria* | *Prolinoborus* | *Tepidicella* |
| *Caulobacteraceae* | *Methylobacterium* | *Propionibacterium* | *Thermicanus* |
| *Cloacibacterium* | *Methylophilaceae* | *Pseudolabrys* | *Thermus* |
| *Comamonadaceae* | *Methylophilales* | *Pseudomonas* | *Variovorax* |
| *Comamonas* | *Methylophilus* | *Ralstonia* | *Vulcanibacillus* |
| *Corynebacterium* | *Methyloversatillis* | *Rhizobiales* |  |
| *Cupriavidus* | *Microbacterium* | *Rhodococcus* |  |
