# Supplementary material for: Bacterial and fungal characterization of pancreatic adenocarcinoma from Endoscopic Ultrasound-guided biopsies
Source: Front Immunol. 2023 Oct 13;14:1268376. doi: 10.3389/fimmu.2023.1268376 (PMC10611524; doi:10.3389/fimmu.2023.1268376)
Supplement: Supplementary file 2 [file Table_2.docx]

**Table S2. Bacteria genera identified exclusively or concomitantly in the FNA and surgical specimens after contaminant removal.**

| **FNA** | **FNA & Surgery** | **Surgery** |
| --- | --- | --- |
| *Acetoanaerobium* | *Actinobacillus* | *Anaerococcus* |
| *Anaerosporobacter* | *Actinobaculum* | *Anaeroglobus* |
| *Anaerovorax* | *Actinomyces* | *Anaerotruncus* |
| *Atopobium* | *Aggregatibacter* | *Asanoa* |
| *Butyricimonas* | *Akkermansia* | *Bibersteinia* |
| *Butyrivibrio* | *Alistipes* | *Centipeda* |
| *Capnocytophaga* | *Bacteroides* | *Citrobacter* |
| *Clostridium_III* | *Barnesiella* | *Clostridium_sensu_stricto* |
| *Clostridium_XI* | *Bifidobacterium* | *Conexibacter* |
| *Clostridium_XVIII* | *Blautia* | *Cronobacter* |
| *Collinsella* | *Campylobacter* | *Desulfovibrio* |
| *Coprococcus* | *Cardiobacterium* | *Dolosigranulum* |
| *Dorea* | *Clostridium_XlVa* | *Enterococcus* |
| *Dysgonomonas* | *Dialister* | *Fibrella* |
| *Hallella* | *Eubacterium* | *Finegoldia* |
| *Klebsiella* | *Faecalibacterium* | *Flavitalea* |
| *Mogibacterium* | *Fusobacterium* | *Geminicoccus* |
| *Oribacterium* | *Gemella* | *Haliangium* |
| *Paraprevotella* | *Granulicatella* | *Jiangella* |
| *Parasporobacterium* | *Haemophilus* | *Leuconostoc* |
| *Pasteurella* | *Lactobacillus* | *Lysobacter* |
| *Planobacterium* | *Leptotrichia* | *Nocardioides* |
| *Porphyromonas* | *Megasphaera* | *Olsenella* |
| *Pseudobutyrivibrio* | *Moraxella* | *Pantoea* |
| *Raoultella* | *Moryella* | *Papillibacter* |
| *Robinsoniella* | *Oscillibacter* | *Peptoniphilus* |
| *Schwartzia* | *Parabacteroides* | *Phytohabitans* |
| *Subdoligranulum* | *Parvimonas* | *Pseudonocardia* |
| *Tannerella* | *Peptostreptococcus* | *Rhodobacter* |
| *Treponema* | *Prevotella* | *Salinibacterium* |
|  | *Roseburia* | *Skermanella* |
|  | *Ruminococcus* | *Sphingobacterium* |
|  | *Selenomonas* | *Sporobacter* |
|  | *Solobacterium* | *Tatumella* |
|  | *Veillonella* |  |
